# A novel multiplex isothermal amplification method for rapid detection and identification of viruses

**DOI:** 10.1038/srep17925

**Published:** 2015-12-08

**Authors:** Dougbeh-Chris Nyan, Kevin L. Swinson

**Affiliations:** 1 Division of Emerging and Transfusion-Transmitted Diseases, Center for Biologics Evaluation and Research, Food and Drug Administration, Silver Spring, MD, USA; 2 Department of Biology, Morgan State University, Baltimore Maryland, USA

## Abstract

A rapid multiplex isothermal amplification assay has been developed for detection and identification of multiple blood-borne viruses that infect millions of people world-wide. These infections may lead to chronic diseases or death if not diagnosed and treated in a timely manner. Sets of virus-specific oligonucleotides and oligofluorophores were designed and used in a reverse-transcription loop-mediated multiplexed isothermal amplification reaction for detection and gel electrophoretic identification of human Immunodeficiency virus (HIV), hepatitis-B virus (HBV), hepatitis-C virus (HCV), hepatitis-E virus (HEV), dengue virus (DENV), and West Nile (WNV) virus infection in blood plasma. Amplification was catalyzed with two thermostable enzymes for 30–60 minutes under isothermal condition, utilizing a simple digital heat source. Electrophoretic analysis of amplified products demonstrated simultaneous detection of 6 viruses that were distinctly identified by unique ladder-like banding patterns. Naked-eye fluorescent visualization of amplicons revealed intensely fluorescing products that indicated positive detection. The test demonstrated a 97% sensitivity and a 100% specificity, with no cross-reaction with other viruses observed. This portable detection tool may have clinical and field utility in the developing and developed world settings. This may enable rapid diagnosis and identification of viruses for targeted therapeutic intervention and prevention of disease transmission.

Blood-borne pathogens such as hepatitis B virus (HBV), hepatitis C virus (HCV), and the emerging hepatitis E virus (HEV) together infect over 800 million people globally and may lead to chronic active hepatitis and hepatocellular carcinoma^1^,^2^,^3^. Infection with the human immunodeficiency virus (HIV) compromises the immune system, while dengue virus (DENV) and West Nile virus (WNV) infections may lead to hemorrhagic fever and neuroinflammatory conditions in infected patients, respectively^4^,^5^.

These viruses are transmitted through blood-borne routes and may co-infect a patient or co-circulate in various geographic regions as indicated by the prevalence of HIV, HCV, HEV, and HBV in North America, Africa, Asia, and Europe. Outbreaks of DENV and WNV have occurred in regions throughout the world, particularly in the Middle East, Europe, Africa, the United States, South America, and the Caribbean^6^,^7^,^8^,^9^,^10^. The global distribution of these pathogens coupled with the clinical manifestations of their disease spectra emphasizes the need for simple, but efficient diagnostic point-of-care tools. Such tools may enable rapid detection and differentiation of viruses that present similar clinical symptoms, aid in targeted therapeutic intervention, and facilitate implementation of medical counter measures for disease control and prevention.

Several amplification-based multiplex methods, including the quantitative reverse-transcription polymerase chain reaction (qRT-PCR) have been developed and used to detect and quantitate pathogen load^11^,^12^. However, these methods exhibit drawbacks as they are time-consuming, expensive, and require the use of heavy equipment and highly trained personnel to perform. Despite their outstanding performance, these characteristics present limitations in their point-of-care use in field and resource-limited environments. Besides, many (reverse transcription) loop-mediated isothermal amplification assays (RT)-LAMP have been designed, but have hardly demonstrated multiplexing capability to simultaneously detect different types of viruses^13^,^14^,^15^,^16^,^17^. A recent study reported a combined multiplex RT-LAMP and immunochromatographic system for colorimetric detection, but this method was confined to the detection and subtyping of only influenza A virus^18^. Other LAMP systems have relied on the use of SYBR Green, GelGreen, calcein or hydroxy naphthol blue for quantitation or visual detection of amplified products, but the dyes are target-independent and non-specific^15^,^17^,^19^,^20^,^21^.

This work describes the development of the first multiplex fluorogenic loop-mediated isothermal amplification assay for simultaneous detection and identification of 6 different viral pathogens in human plasma. This assay is based on fluorogenic hybridization and autocycling strand-displacement DNA synthesis that is loop-mediated under isothermal amplification conditions and produce repeats of nucleic acid amplicons as ladder-like banding patterns^13^,^14^,^15^,^16^,^17^,^22^. The method utilizes 3 pairs of virus-specific primers (including target-specific fluorogenic oligonucleotides) that target 8 different sequences within the genomic region of interest (Table 1; Fig. 1). The specific fluorogenic oligonucleotides hybridize only to the target-sequence of the amplified DNA, resulting in emission of fluorescence that is visible to the naked-eye under UV-illumination and measureable with a fluorospectrophotometer. The amplification process is accomplished within 30–60 minutes, utilizing a portable digital heating source. Here, we present a simple diagnostic approach that have clinical application for rapid detection and simultaneous identification of multiple viral pathogens.

## Results

### Detection of viral nucleic acid

We applied standards of viral nucleic acids to isothermal multiplexed amplification reaction in order to evaluate the ability of the assay to detect multiple viral targets. Electrophoretic analysis of reaction products revealed amplification of nucleic acids of HIV, HBV, HCV, HEV, DENV and WNV. Positive amplification was demonstrated by the presence of specific ladder-like banding patterns. No banding-pattern was observed for the no-template control (NTC), parvovirus (PV), and cytomegalovirus (CMV) which served as negative controls (Fig. 2A; see Supplementary Figs S1-S4 online).

### Fluorescence detection

All virus-specific fluorogenic oligonucleotides were used in a multiplex amplification reaction mixture to enable visual detection of amplified viral nucleic acids and facilitate measurement of fluorescence intensities. The reaction products were visualized under UV-illumination and revealed an intense fluorescence in tubes with amplified products (HIV, HBV, HCV, HEV, DENV, and WNV), but no glow was observed in the negative control tubes (Fig. 2B). Spectrofluorometric measurement of the same products revealed higher relative fluorescence units (RFU) in the amplified target as compared with the negative controls (Fig. 2B).

### Analytical sensitivity

Dilutions of all target nucleic acids, including HBV-DNA standards ranging from 10^4^–25 IU/reaction and HCV-RNA standards ranging from 10^3^–10 IU/reaction (IU/rxn) were used in amplification reaction to investigate the analytical sensitivity of the assay. Results of electrophoretic gel analysis revealed detection of 50 IU/rxn of HBV-DNA and 10^2^ IU/rxn of HCV-RNA (Fig. 3, Panel-1A and Panel-2A); evaluation of assay sensitivity using the other targets revealed a detection limit detection of approximately 10^2^–50 viral particles per reaction. Spectrofluorometric measurement revealed higher RFU of HBV-DNA serial dilutions 50 IU/rxn as compared with the negative controls (Fig. 3, Panel-1B and 1C; Table 2A). Interestingly, the 25 IU HBV-DNA dilution revealed higher RFU than the controls, but reacted negative on gel electrophoresis. Similarly, the 10^2^ and 10 IU/rxn of HCV-RNA showed higher RFUs as compared with the negative controls, but the 10 IU/rxn dilution showed no banding pattern on agarose gel electrophoresis (Fig. 3, Panel-2B and 2C; Table 2B).

### Analytical specificity

Primer sets and fluorogenic oligonucleotides were tested against nucleic acids of HCV, HIV, HBV, DENV, HEV, WNV, CMV, and PV in the multiplex isothermal amplification assay in order to investigate the analytical specificity. Electrophoretic analysis of reaction products demonstrated amplification of the targeted viral pathogens by their specific oligonucleotide-sets and produced distinctive ladder-like banding patterns for each detected virus (Figs 2A and 4A all panels; see Supplementary Figs S1-S4 online). Also, UV analysis revealed that the virus-specific fluorogenic oligonucleotides hybridized only to their target nucleic acids as demonstrated by the intense fluorescence of the amplified samples and compared with the controls and non-targets (Fig. 4B all panels).

### Fluorimetric analysis of reaction products

Spectrofluorometric measurement of reaction products was performed in order to quantify the fluorescence emitted by the amplified targets as compared with the reaction negative controls. Analysis of the results revealed higher RFU values for the detected targets, but lower RUFs for the negative controls (Figs 2B, 3B,C-all panels, and 4B-all panels). In Fig. 5, the two specific fluorogenic oligonucleotides that were employed for detection of an HIV-HCV co-infection successfully hybridized with their targets. This was demonstrated by the higher RFUs of the HCV and HIV duplicate samples, while lower RFUs were observed for the non-target nucleic acids.

### Test of clinical specimens

Nucleic acids were extracted from 89 clinical plasma specimens, including HIV, HBV, HCV, WNV, CMV, DENV, PV, and healthy human plasma (see Supplementary Table S1 online). The DNA and RNA extracts were tested to validate the clinical performance of the multiplex isothermal assay. Results of amplification showed that the assay detected 36 out of 37 infected clinical specimens, thereby demonstrating a 97% diagnostic sensitivity. The results further revealed that all 52 assay-controlled plasma specimens reacted negative, thus demonstrating a 100% diagnostic specificity (Table 3).

## Discussion

We have developed a rapid fluorogenic multiplex isothermal amplification assay that simultaneously detects and identifies different viral pathogens. The assay utilized a reaction master mixture that simultaneously amplified both DNA and RNA, and specifically amplified RNA in a one-step single-tube procedure. In addition, this method provides three end-point read-outs, utilizing distinctive gel-electrophoretic banding patterns for viral differentiation, measurement of fluorescence intensities, and naked-eye fluorescence visualization for rapid detection (Figs 2, 3, 4, 5). Furthermore, the assay demonstrated an analytical sensitivity of 10^2^ IU/rxn using RNA and 50 IU/rxn utilizing DNA. The assay also revealed a specificity characterized by amplification of only the viruses that were targeted for detection. For example, the specific-primer sets only detected HIV, HBV, HCV, HEV, DENV, and WNV, but reacted negative to PV and CMV (Figs 2 and 4). For assay validation, nucleic acids extracts from clinical donor plasma specimens were used in amplification reaction (see Supplementary Table S1 online). As shown in Table 3, the assay revealed a plausible diagnostic sensitivity of 97% and a 100% diagnostic specificity when compared with the FDA-approved Procleix test. These diagnostic characteristics point to the potential applicability of the assay for blood screening and clinical point-of-care diagnostic use (Table 3).

**Figure 1:**
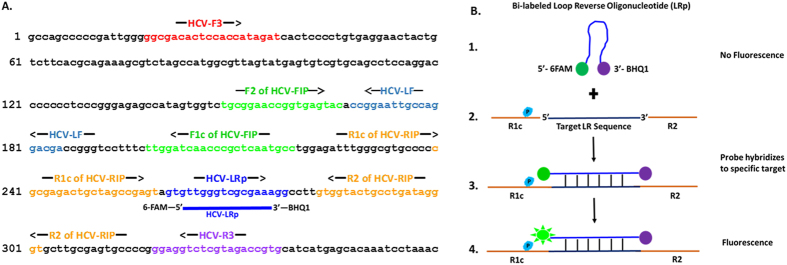
Position of oligonucleotides, oligofluorophores hybridization, and mechanism of detection illustrated on the HCV 5′-NCR. **(A)** Illustration of the positions of oligonucleotides and oligofluorophores used for sequence-specific detection on the 5′-NCR of the HCV genome. **(B)** Biomolecular mechanism of the bi-labeled Loop Reverse oligonucleotide (LRp) covalently linked with 6-FAM as reporter on the 5′ end and BHQ1 as quencher on the 3′end. In step 1, in the absence of hybridization to amplified product (target sequence), the reporter remains quenched, with no fluorescence emission due to the close proximity of the quencher to the fluorophore. In the presence of amplified product in steps 2 and 3, the LRp hybridizes to the specific target between the R1c and R2 segments of the gene sequence, thus fluorophore begins to fluoresce as it separates from the quencher. From step 3 to step 4 as Bst DNA Polymerase catalyzes the reaction, quenching is disrupted and fluorescence is increased due to the increased distance (separation) between the reporter and quencher when the fluorooligonucleotide hybridizes and also due to fluorescence resonance energy transfer (FRET). The emitted fluorescence which corresponds to the amount of amplified nucleic acid is measured by spectrofluorometer in relative fluorescence units (RFU) and visualized by the naked-eye under UV-illumination.

**Figure 2:**
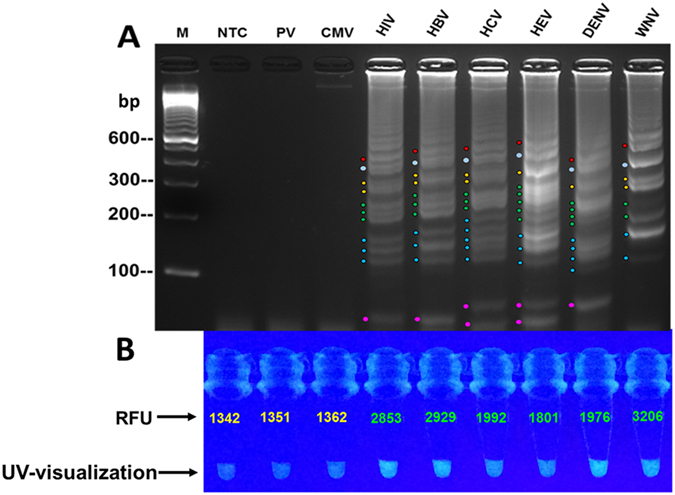
Multiplex detection and identification of viruses. Standard reference viral nucleic acids were subjected to isothermal multiplex amplification to evaluate the assay’s detection ability utilizing virus-specific primers-sets and specific fluorogenic oligonucleotides. Detected targets were identified by banding-pattern analysis. Targets were amplified with a master reaction mixture containing all the primer-sets and all the 6-FAM/BHQ1-tagged fluorogenic oligonucleotides. Differences in banding patterns were observed and identified by the location of pattern formation in relation to the molecular marker, size of the bands within the patterns, spacing of each group of patterns and the laddering-shift of the patterns (also see Supplementary Figures S1-S4). **(A)** Electrophoretic analysis showed detection of all target nucleic acids with distinguishing ladder-like banding patterns in lanes 4–9 for human Immunodeficiency virus (HIV), hepatitis-B virus (HBV), hepatitis-C virus (HCV), hepatitis-E virus (HEV), dengue virus (DENV), and West Nile (WNV). No detection was observed in the negative controls which includes the non-template control (NTC in lane 1), cytomegalovirus (CMV in lane 2), and, parvovirus (PV in lane 3); M = 100 bp marker. Note that for WNV distinct pattern generation, W-LF may not be required in reaction. **(B)** Fluorimetric investigation revealed positive fluorescence detection of HIV, HCV, HBV, HEV, DENV, and WNV as demonstrated by high fluorescence intensity and higher relative fluorescence unit (RFU) as compared with the NTC, CMV, and PV controls.

Detection systems such as quantitative reverse-transcription polymerase chain reaction (qRT-PCR) employ a single pair of primer and a probe for detection of targets. In contrast, we used 3 pairs of pathogen-specific primers (including an oligofluorophore) that target 8 different sequences on the genomic region of interest (Fig. 1A). In this study, we designed target-specific Loop Reverse oligonucleotides (LRp) of each primer set as fluorogenic probes covalently linked with either 6-FAM, Tet, or Texas-Red as reporter on the 5′ end and Black Hole Quenchers (BHQ) on the 3′end (Fig. 1B). As illustrated in Fig. 1, the fluorogenic oligonucleotides hybridize only to the virus-specific sequence located between the R1c and R2 segments of the targeted gene. During strand displacement DNA synthesis by *Bst* DNA Polymerase, the reporter-fluorophore at the 5′end of the fluorogenic oligonucleotide is separated from the quencher at the 3′end by the increase in distance upon hybridization. An intense fluorescence is emitted and measured by a spectrofluorometer or visualized by the naked-eye (Figs 1B, 2B, 4B-all panels, and 5). The biomolecular mechanism used in this study ensured amplification efficiency and detection specificity.

Each virus of interest was detected on agarose gel due to the presence of all the primer-sets in the multiplex master reaction-mixture. The distinguishing ladder-like banding-patterns generated by each primer-set then enabled pathogen identification (Figs 2A and 4A-all panels; see Supplementary Figs S1-S4 online). The differences in banding patterns are identifiable by the group-formation of the bands relative to the molecular marker, the size of the bands within the patterns, and position or laddering-shift of the detected targets as illustrated by the color lines and dots (Figs 2A and 4; see Supplementary Figs S1-S4 online). Like products of other amplification platforms, gel electrophoresis of RT-LAMP products does risk cross-contamination by amplified DNA. Precautions to avoid this and the use of negative (no-template) control assays are essential to identify contamination problems and monitor assay integrity. Furthermore, upon post-amplification UV-visualization of reaction tubes, only the amplified targets produced intense fluorescence (Fig. 4B-all panels and 5). The results of this biomolecular process suggest that the oligofluorophores hybridized only to the specific viral nucleic acids without cross-reacting with nucleic acids of the non-target viruses. This approach obviated the post-amplification addition of DNA intercalating dyes such as SYBR Green and GelGreen or the use of metal indicator, calcein which are non-specific indicators of amplified DNA^15^,^17^,^19^,^20^,^21^. On the contrary, the fluorogenic oligonucleotides used in our study are specific; hybridization to their targets were confirmed by the higher RFUs and increased fluorescent intensity of the detected targets as compared with the non-target viruses. (Fig. 4B-all panels and 5).

**Figure 3:**
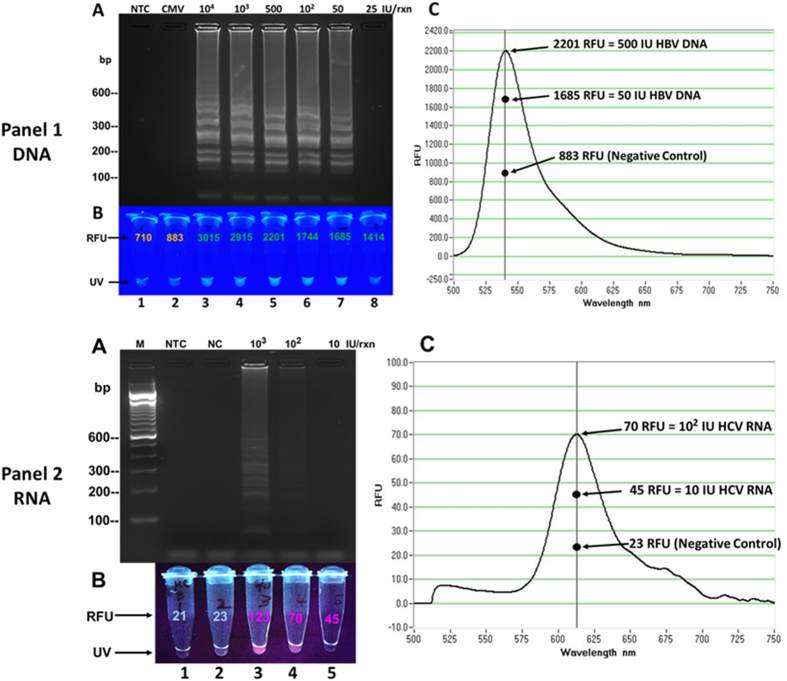
Assay sensitivity and quantitative evaluation using DNA and RNA. Assay detection sensitivity and fluorescent measurement was investigated using serial dilutions of hepatitis-B virus (HBV) DNA and hepatitis C virus (HCV) RNA. Amplification was performed utilizing the TET/BHQ1-tagged fluorogenic oligonucleotide of HBV DNA and the Texas-Red/BHQ2-tagged fluorogenic oligonucleotide for HCV RNA. **(A)** Results revealed assay detection of 50 IU/rxn of HBV DNA (Panel 1, lane 7) and 10^2^ IU/rxn of HCV RNA (Panel 2, lane 4) as confirmed by the presence of banding pattern. **(B)** Fluorimetric measurement revealed higher RFUs of HBV DNA serial dilutions in tubes 3–8 as compared with the controls in tubes 1 and 2 (Panel 1) and higher RFUs of HCV RNA dilutions in tubes 3–5 as compared with the controls in tubes 1 and 2 (Panel 2). Note the absence of banding patterns, but higher RFU in tube 8 (panel 1) and tube 5 (panel 2). **(C)** Graphs showed fluorescence detection peak of amplified products in both panels. RFU = Relative Fluorescence Units; UV = ultraviolet naked-eye visualization.

**Figure 4:**
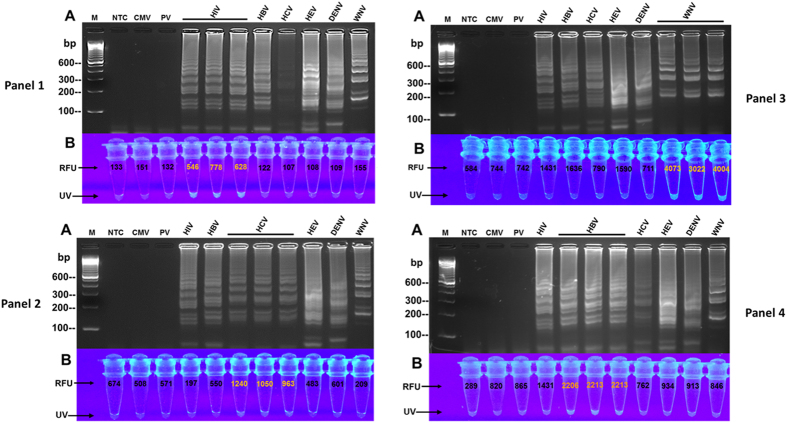
Detection specificity of primers and fluorogenic oligonucleotides. Ability of the assay to detect specific targets was evaluated utilizing nucleic acid extracts of clinical specimens and multiplex master reaction mixture containing all the primer-sets in Table 1 and only one virus-specific fluorogenic oligonucleotide (LRp) per panel. **(A)** Electrophoretic results in all 4 panels show detection of the targets as demonstrated by virus-specific ladder-like banding-patterns. No banding pattern was observed in the controls, NTC, CMV, and PV. Distinguishing characteristics of the banding patterns were identified by the grouping/pattern-formation of the bands, size and number of the bands in each pattern, and spacing/distance between the patterns (also see Supplementary Figure S4 for target identification). **(B)** Intense fluorescence and higher RFU demonstrate that: (i) HIV-LRp specifically hybridized only to HIV RNA in Panel-1; (ii) HCV-LRp specifically hybridized only to HCV RNA in Panel-2; (iii) WNV-LRp specifically hybridized only to WNV RNA in Panel-3; and, (iv) HBV-LRp specifically hybridized only to HBV DNA in Panel-4. M = 100 bp marker; NTC = non-template control; CMV = cytomegalovirus; PV = parvovirus; Human Immunodeficiency virus (HIV); Hepatitis-B virus (HBV); Hepatitis-C virus (HCV); Hepatitis-E virus (HEV); Dengue virus (DENV); West Nile virus (WNV); RFU = Relative Fluorescence Units; UV = ultraviolet naked-eye visualization.

Fluorophores have been traditionally used in immunohistochemistry for identification of cell structures and in qPCR applications for detection and quantitation of pathogen load^22^,^23^. The properties of these fluorogenic dyes to absorb and emit light or transfer energy were employed by this study for detection and measurement of the fluorescence of amplified viral nucleic acid. In this study, we successfully utilized three different fluorophores (Tet, 6-FAM, and Texas-Red) for detection. Furthermore, we observed that amplified nucleic acid demonstrated higher RFUs that indicated positive detection, while the non-target viral nucleic acids and negative controls revealed lower RFUs. For example, the results of the assay sensitivity test showed a visual and quantitative increase in fluorescence intensity of the HBV-DNA and HCV-RNA dilutions as compared with the negative control samples (Fig. 3; Table 2). Also, viruses that were detected by their specific oligofluorophores produced higher RFU values, while the non-targeted viruses and negative controls revealed lower RFU values (Fig. 4B-all panels). In this study, we have consistently observed that the increase fluorescence intensity corresponds to amplification of the target nucleic acid. Therefore, we propose that this biomolecular process could potentially be used for rapid empirical quantitation of amplified nucleic acid as this could correspond to pathogen load in an infected specimen. However, additional studies are warranted.

**Figure 5:**
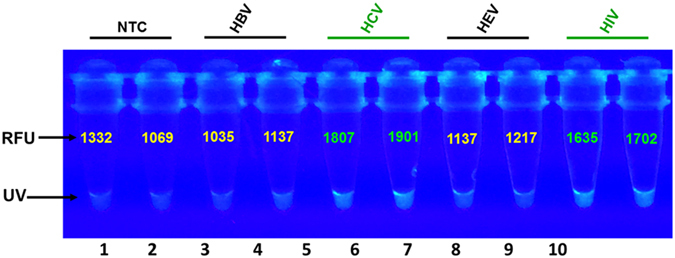
Co-detection of viruses with specific oligofluorophores. Two fluorogenic oligonucleotides (6-FAM/BHQ1-HIVLRp and 6-FAM/BHQ1-HCV-LRp) were simultaneous applied in multiplex reaction containing all the primers in Table 1 for detection of only hepatitis C virus (HCV) and human Immunodeficiency virus (HIV) nucleic acid extracts. Fluorospectrophotometric results demonstrate brighter fluorescence and higher RFUs in the HCV samples (lanes 5 and 6) and the HIV (lanes 9 and 10) as compared with the no-template controls (NTCs) in lanes 1 and 2, hepatitis B virus (HBV) in lanes 3 and 4, and hepatitis E virus (HEV) in lanes 7 and 8; UV = ultraviolet naked-eye visualization.

In view of the prevalence of co-infection of the hepatitis viruses and HIV particularly among injection drug users (IUDs)^5^, we simulated a test for detecting a co-infection of this kind. When two specific oligofluorophores targeting HCV-RNA and HIV-RNA were simultaneously utilized in the amplification reaction mixture, the results revealed detection of only the two target-viruses as observed in Fig. 5. The specificity of this co-detection was confirmed by the intense fluorescence of the amplified targets. These amplified specimens also produced higher RFUs as compared with the RFUs of the controls and non-target samples (Fig. 5). Although the detection characteristics of this assay have proven relevant to clinical applications, we believe that additional optimization and improvements could further enhance its capabilities. Efforts are underway in this endeavor, including plans for a large-scale field trial.

The data reported in this study have demonstrated the potential application of this assay for simultaneous detection, identification, and measurement of amplified products of multiple viral targets. The assay revealed a plausible diagnostic sensitivity and specificity. Taken together, these results suggest that the assay could be useful for point-of-care diagnostics, donor blood screening, and investigating the epidemiological trend of viral pathogens in resource-limited communities of the developed and developing world settings.

## Methods

### Nucleic acid standards

DNA and RNA were extracted from HBV, HIV, HEV, and HCV reference plasma panels of WHO International Standard (OptiQuant-AcroMetrix/Life Technology, Benicia, CA, USA and Sera Care, Milford, MA, USA). Extraction was performed using the QiaAmp Viral RNA mini-kit and the QiAamp DNA Blood mini-kit according to manufacturer’s protocol with some modifications (Qiagen, Germantown, MD, USA). HEV standard was a kind gift of Dr. Suzanne Emmerson (National Institutes of Health, Bethesda, Maryland). Armored RNA standards of HIV, DENV, WNV, and HCV were purchased from Asuragen (Austin, TX, USA).

### Oligonucleotides and probes design

Several full-length sequences of various viral pathogens (e.g. HIV, HBV, HCV, HEV, WNV, and DENV) were obtained from the GenBank database and analyzed using CLUSTALW-2. Conserved genetic regions and selected sequences were targeted for primer design (Table 1). A set of primers containing 3 pairs of pathogen-specific oligonucleotides (including bi-labeled fluorogenic oligonucleotides) were manually designed for each viral target. Primer sets consisted of the following: Forward Inner Primer (FIP); Reverse Inner Primer (RIP); Loop Forward Primer (LF); Loop Reverse Primer (LR); Forward Outer Primer (F3), and Reverse Outer Primer (R3). The two sequences of FIP (F1c and F2) and RIP (R1c and R2) were spaced with “TTTT” linker. The Loop Reverse Primers (LR) were designed as fluorogenic oligonucleotide conjugated with either 6-Carboxyfluorescein (6-Fam), Tetrachlorofluorescein (Tet) or Texas-Red at the 5′-end and Black Hole Quenchers (BHQ) at the 3′-end (Table 1, Fig. 1). All oligonucleotides and oligofluorophores were synthesized by EuroFins MWG Operon (Huntsville, AL, USA) and Integrated DNA Technologies (Coralville, IA, USA).

### Multiplex isothermal amplification assay

Amplification of viral DNA and RNA was conducted as a one-step reaction by strand-displacement (reverse-transcription) loop-mediated isothermal amplification in a 25 μL reaction mixture. The reaction master mixture comprised of 12.5 μL of Mannitol Acetates Buffer (MAB) previously described^17^. The master mixture also consisted of all the virus-specific oligonucleotide sets and the specific fluorooligonucleotides of intended targets. Optimal primers and oligofluorophores concentrations were as follows: 1.0 μM each of primers FIP and RIP, 0.6 μM each of primers LF and LR, 0.4 μM each of primers F3 and R3, 0.3 μM of 6-FAM/BHQ1, 0.3 μM of Texas-Red/BHQ2, and 0.8 μM of TET/BHQ1 labeled oligofluorophores. Five units of RNaseOut, 5 U of cloned AMV reverse-transcriptase (Invitrogen, Frederick, USA), and 8 Units of *Bst* DNA polymerase (New England Biolabs, MA, USA) were also added to the reaction mixture. Viral RNA and DNA template-volume of 5 μL of both target and assay-controlled viruses were added to the amplification reactions. A no-template (water) control and nucleic acid extracted from normal human plasma were included in amplification runs as controls. Reaction reagents were prepared in a PCR work-station (Plas-Lab, MI, USA), with precautionary measures observed to prevent cross-contamination. Amplification-reactions were conducted under isothermal condition at 60 °C for 60 minutes on a portable heating device. Reactions were terminated by placing reaction tubes on ice.

### Fluorescence detection and spectrofluorometric analysis of products

In order to investigate as to whether the virus-specific fluorogenic oligonucleotides hybridized to their targets, fluorescence intensities in reaction tubes were evaluated with the naked-eye using a handheld UV-transilluminator at 302 nm. Reaction tubes were photographed using a BlackBerry Z30 smartphone camera (Research-In-Motion, Canada) and the iPad Air tablet camera (Apple, Inc., USA). Additionally, fluorimetric evaluation of fluorescence intensity was performed by measuring 1.5 μL of target and control amplification products on a NanoDrop-3300 spectrofluorometer (ThermoScientific, DE, USA).

### Electrophoretic evaluation of amplification products

To confirm amplification and evaluate the different ladder-like banding patterns of the targets, 5 μL of (RT)-LAMP reaction products were electrophoresed on 2.8% agarose gels. Gels were prepared in 1x Tris-borate EDTA buffer (TBE) and contained 10x GelRed DNA intercalating dye (Phenix Research, Candler, NC). Electrophoretic pattern differences were observed and identified by the location and formation of banding-patterns in relation to the molecular marker, size of the bands within the patterns, spacing of each group of patterns as well as the laddering-shift of the patterns of detected targets. Gels were visualized under UV-transilluminator at 302 nm and photographed with the GBOX gel documentation system (Syngene, Frederick, MD, USA).

### Assay specificity and sensitivity

In order to investigate the specificity of the assay, primer sets and fluorogenic oligonucleotides were tested against nucleic acids of HCV, HIV, HBV, DENV, HEV, WNV, CMV, and PV in the multiplex isothermal amplification assay. In order to determine the assay’s limit of detection, dilutions of nucleic acids of various targets were evaluated. Sensitivity tests representative of DNA and RNA viruses were performed by testing dilutions of HBV DNA standards ranging from 10^4^–25 IU/rxn and HCV RNA standards ranging from 10^3^–10 IU/rxn.

### Test of clinical plasma specimens

A total of 89 clinical donor specimens that were randomly pre-selected with the FDA-approved Procleix test^23^ (and packet insert) were used to investigate the clinical utility of the isothermal multiplex assay in comparison to the Procleix test: 52 non-target control specimens, including healthy human plasma and clinical donor plasma specimens infected by cytomegalovirus (CMV) plasma and parvovirus (PV) were tested; 37 targeted clinical specimens, including donor plasma from patients infected by HIV, WNV, DENV, HBV, and HCV were also tested (see Supplementary Table S1 online).

## Additional Information

**How to cite this article**: Nyan, D.-C. and Swinson, K. L. A novel multiplex isothermal amplification method for rapid detection and identification of viruses. *Sci. Rep.* **5**, 17925; doi: 10.1038/srep17925 (2015).

## Supplementary Material

Supplementary Information

## Figures and Tables

**Table 1:** Oligonucleotide and oligoprobe sequences used for multiplex isothermal amplification and detection.

Virus and primer set	Primer name	Primer Sequence (5′-3′)	Genetic region	Accession number
HIV	HIV-F3	ACACAGTGGGGGGACATCAAGC	GAG	JQ416161
	HIV-R3	TCCATGCTATTTGTTCCTGAAGGG		
	HIV-FIP	GATGCAATCTATCCCATTCTG-TTTT-GCCATGCAAATGTTAAAAG		
	HIV-RIP	AGTCCATGGAGGGCCTATTGCAC-TTTT- GTTCCTGCTATGTCACTTCC		
	HIV-LF	CAGCTTCCTCATTGATGGTCT		
	HIV-LRp	6-Fam-CAGGCCAGATGAGAGAACCAA-BHQ1		
^£^HBV	HBU-F3	TCCTCACAATACCGCAGAGT	P and S gene	AB116094
	HBU-R3	GCAGCAGGATGAAGAGGAAT		
	HBU-FIP	GTTGGGGACTGCGAATTTTGGC-TTTT-TAGACTCGTGGTGGACTTCT		
	HBU-RIP	TCACTCACCAACCTCCTGTCCT-TTTT-AAAACGCCGCAGACACAT		
	HBU-LF	GGTGATCCCCCTAGAAAATTGAG		
	HBU-LRp	Tet-AATTTGTCCTGGTTATCGCTGG-BHQ1 6-FAM-AATTTGTCCTGGTTATCGCTGG-BHQ1		
HCV	HCV-F3	GGCGACACTCCACCATAGAT	5′NCR	AF333324
	HCV-R3	CACGGTCTACGAGACCTCC		
	HCV-FIP	GGCATTGAGCGGGTTGATCCAA-TTTT-TGCGGAACCGGTGAGTAC		
	HCV-RIP	CGCGAGACTGCTAGCCGAGT-TTTT-ACCCTATCAGGCAGTACCAC		
	HCV-LF	TCGTCCTGGCAATTCCGG		
	HCV-LRp	6-FAM-GTGTTGGGTCGCGAAAGG-BHQ1 Texas-Red-GTGTTGGGTCGCGAAAGG-BHQ2		
HEV	HEV-F3	CGGCGGTGGTTTCTGGGGTGACA	ORF2/3	AB437318
	HEV-R3	GAGATAGCAGTCAACGGCGC		
	HEV-FIP	AGGGCGAGCTCCAGCCCCGG-TTTT-GCCCTTCGCCCTCCCCTATATT		
	HEV-RIP	CCAGTCCCAGCGCCCCTCCG-TTTT-AGCTGG GGCAGA TCGACGAC		
	HEV-LF	ATTGTGAAACGACATCGGCG		
	HEV-LRp	6-FAM-TCGTCGATCTGCCCCAGCT-BHQ1		
DENV	D-F3	AGCTTCATCGTGGGGATGT	3′NCR	M87512
	D-R3	CTCTCCCAGCGTCAATATGC		
	D-FIP	GGAGGGGTCTCCTCTAACCACTTTTTGGCTGCAACCCATGGAAG		
	D-RIP	CAAAACATAACGCAGCAGCGGGTTTTGGGGGTCTCCTCTAACCTC		
	D-LF	TGCTACCCCATGCGTACAG		
	D-LRp	6-FAM-CAACACCAGGGGAAGCTGT-BHQ1		
WNV	W-F3	CGATTTGTGTTGGCTCTCTTGGCGT	5′NCR	JN183893
	W-R3	AGGCCAATCATGACTGCAAT		
	W-FIP	CTCTCCATCGATCCAGCACTGCTTTTCTTGGCGTTCTTCAGGTTCA		
	W-RIP	ACTAGGGACCTTGACCAGTGCTTTTTTCCGGTCTTTCCTCCTCTT		
	W-LF	CGG GTC GGA GCA ATT GCTG		
	W-LRp	6-Fam-TCAATCGGCGGAGCTCAAAAC-BHQ1		

^£^HBV primer set from Nyan *et al.* 2014.

**Table 2:** 

Sample ID	Concentration (IU/rxn)	Quantitative RFU values (at λ 540)	Fluorogenic Detection	Electrophoretic Detection
A. Fluorimetric evaluation of sensitivity of multiplex assay with DNA				
1. NTC	NA	710	−	−
2. CMV	NA	883	−	−
3. HBV-DNA	10^4^	3015	+	+
4. HBV-DNA	10^3^	2915	+	+
5. HBV-DNA	500	2201	+	+
6. HBV-DNA	10^2^	1744	+	+
7. HBV-DNA	50	1685	+	+
8. HBV-DNA	25	1414	+	−
**Sample ID**	**Concentration (IU/rxn)**	**Quantitative RFU values (at λ 613)**	**Fluorogenic Detection**	**Electrophoretic Detection**
1. NTC	NA	21	−	−
2. NC	NA	23	−	−
3. HCV-RNA	10^3^	123	+	+
4. HCV-RNA	10^2^	70	+	+
5. HCV-RNA	10	45	+	−

Spectrofluorometric evaluation of multiplex assay sensitivity. Abbreviations: NTC, no template control; CMV, cytomegalovirus; NC, negative control; HBV, hepatitis B virus; HCV, hepatitis C virus; RFU, relative fluorescence unit; IU/rxn, international units per reaction; NA, not applicable; Note: TET/BHQ1 used for DNA and Texas-Red/BHQ2 used for RNA detection.

**Table 3:** Clinical donor specimens tested by Isothermal Multiplex Assay.

Specimens	Number Tested	Isothermal Multiplex Assay	^¶^Procleix test
A. Negative Clinical specimens (n = 52)		Diagnostic Specificity (Test-Negative)	
Normal Human Plasma	28	28	28
Cytomegalovirus	15	15	15
Parvovirus	9	9	9
Total	52	52	52
Percent Negative		100%	100%
B. Positive Clinical Specimens (n = 37)		Diagnostic Sensitivity (Test-Positive)	
Human immunodeficiency virus	6	5	6
Hepatitis B virus	9	9	9
Hepatitis C virus	10	10	10
Dengue virus	5	5	5
West Nile virus	7	7	7
Total	37	36	37
Percent Positive		97%	100%

^**¶**^Procleix data based on selected cohort; Abbreviation: NA, not applicable.
