# Supplementary material for: A novel multiplex isothermal amplification method for rapid detection and identification of viruses
Source: Sci Rep. 2015 Dec 8;5:17925. doi: 10.1038/srep17925 (PMC4672323; doi:10.1038/srep17925)
Supplement: Supplementary Information [file srep17925-s1.pdf]

# A novel isothermal amplification method for rapid detection and identification of viruses

## Supplementary Information

***[1]Dougbeh-Chris Nyan and [2]Kevin L. Swinson**

[1]Division of Emerging and Transfusion-Transmitted Diseases, Center for Biologics Evaluation and Research, Food and Drug Administration, Silver Spring, MD, USA;

[2]Department of Biology, Morgan State University, Baltimore Maryland, USA

***Corresponding Author:** Dougbeh-Chris Nyan, M.D. (dnyan@doctor.com; Tel: 240-421-5909); Food and Drug Administration (FDA), 10903 New Hampshire Ave, Silver Spring, MD 20993.

# Supplementary Table and Figures

**Table S1. Clinical donor specimens tested**

| Clinical Specimens | Test Results | |
|---|---|---|
| | Multiplex Isothermal Method | Procleix Test |
| 1. Normal Human Plasma | – | – |
| 2. Normal Human Plasma | – | – |
| 3. Normal Human Plasma | – | – |
| 4. Cytomegalovirus | – | – |
| 5. Cytomegalovirus | – | – |
| 6. Cytomegalovirus | – | – |
| 7. Cytomegalovirus | – | – |
| 8. Parvovirus | – | – |
| 9. Parvovirus | – | – |
| 10. Parvovirus | – | – |
| 11. Parvovirus | – | – |
| 12. Normal Human Plasma | – | – |
| 13. Normal Human Plasma | – | – |
| 14. Normal Human Plasma | – | – |
| 15. Parvovirus | – | – |
| 16. Parvovirus | – | – |
| 17. Cytomegalovirus | – | – |
| 18. Cytomegalovirus | – | – |
| 19. Parvovirus | – | – |
| 20. Parvovirus | – | – |
| 21. Cytomegalovirus | – | – |
| 22. Cytomegalovirus | – | – |
| 23. Cytomegalovirus | – | – |
| 24. Normal Human Plasma | – | – |
| 25. Normal Human Plasma | – | – |
| 26. Normal Human Plasma | – | – |
| 27. Normal Human Plasma | – | – |
| 28. Cytomegalovirus | – | – |
| 29. Cytomegalovirus | – | – |
| 30. Cytomegalovirus | – | – |
| 31. Cytomegalovirus | – | – |
| 32. Cytomegalovirus | – | – |
| 33. Cytomegalovirus | – | – |
| 34. Parvovirus | – | – |
| 35. Normal Human Plasma | – | – |
| 36. Normal Human Plasma | – | – |
| 37. Normal Human Plasma | – | – |

| | | |
|---|---|---|
| 38. Normal Human Plasma | – | – |
| 39. Normal Human Plasma | – | – |
| 40. Normal Human Plasma | – | – |
| 41. Normal Human Plasma | – | – |
| 42. Normal Human Plasma | – | – |
| 43. Normal Human Plasma | – | – |
| 44. Normal Human Plasma | – | – |
| 45. Normal Human Plasma | – | – |
| 46. Normal Human Plasma | – | – |
| 47. Normal Human Plasma | – | – |
| 48. Normal Human Plasma | – | – |
| 49. Normal Human Plasma | – | – |
| 50. Normal Human Plasma | – | – |
| 51. Normal Human Plasma | – | – |
| 52. Normal Human Plasma | – | – |
| | | |
| 53. Dengue virus | + | + |
| 54. Dengue virus | + | + |
| 55. West Nile virus | + | + |
| 56. West Nile virus | + | + |
| 57. West Nile virus | + | + |
| 58. Dengue virus | + | + |
| 59. Dengue virus | + | + |
| 60. West Nile virus | + | + |
| 61. West Nile virus | + | + |
| 62. Hepatitis C virus | + | + |
| 63. Hepatitis C virus | + | + |
| 64. Hepatitis C virus | + | + |
| 65. Hepatitis B virus | + | + |
| 66. Hepatitis B virus | + | + |
| 67. Hepatitis C virus | + | + |
| 68. Human immunodeficiency virus | + | + |
| 69. Human immunodeficiency virus | + | + |
| 70. Human immunodeficiency virus | – | + |
| 71. Hepatitis B virus | + | + |
| 72. Hepatitis B virus | + | + |
| 73. Hepatitis B virus | + | + |
| 74. Hepatitis B virus | + | + |
| 75. Hepatitis B virus | + | + |
| 76. Hepatitis B virus | + | + |
| 77. Human immunodeficiency virus | + | + |
| 78. Hepatitis C virus | + | + |
| 79. Hepatitis C virus | + | + |
| 80. West Nile virus | + | + |
| 81. Dengue virus | + | + |
| 82. West Nile virus | + | + |

| | | |
|---|---|---|
| **83. Hepatitis C virus** | + | + |
| **84. Human immunodeficiency virus** | + | + |
| **85. Human immunodeficiency virus** | + | + |
| **86. Hepatitis B virus** | + | + |
| **87. Hepatitis C virus** | + | + |
| **88. Hepatitis C virus** | + | + |
| **89. Hepatitis C virus** | + | + |

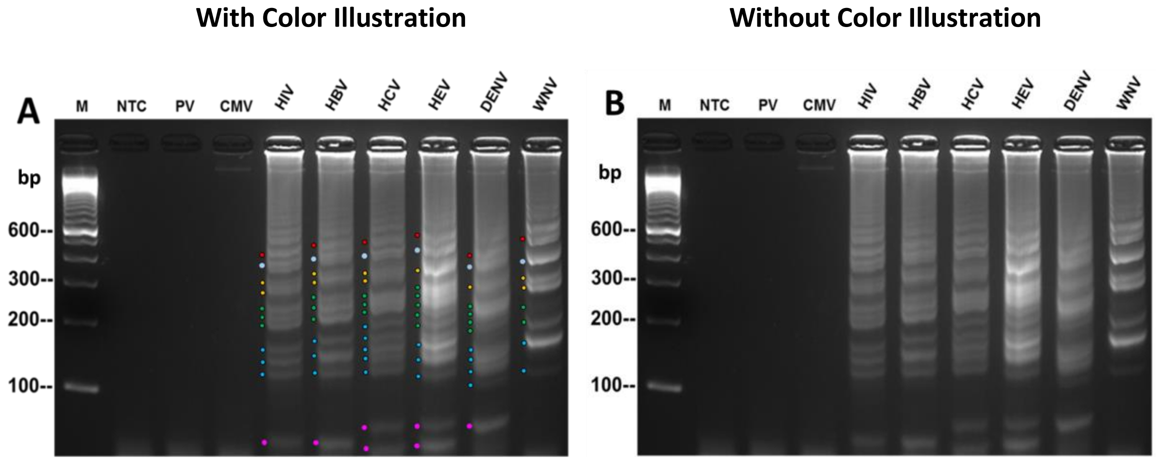


**Supplementary Figure S1. Identification of amplified targets by banding-pattern. (A)** Electrophoretic results showing distinctive ladder-like banding-patterns of amplified targets (HIV, HBV, HCV, HEV, DENV, and WNV). The group of color dots illustrate different grouping of bands, pattern formation, number and sizes of bands, and space between patterns between molecular base pairs 300 and 100 or below. For example, the bands and pattern in the "blue-dotted" area illustrate that HCV has 5 bands, HBV and HIV have 3 each, the 3 bands of HBV are spaced out than those of HIV. The bands and patterns in the "green-dotted" area show that HEV has about 5 bands in a pattern that extends far above the 4 bands of DENV. **(B)** Identical gel showing banding patterns without color illustration. Gel was run for (circa 55 minutes) in order to retain the differentiating bands below bp 100 (pink-dotted). Those are actual bands (not primer-dimers). M = 100 bp marker; NTC = non-template control; CMV = cytomegalovirus; PV = parvovirus; Human Immunodeficiency virus (HIV); Hepatitis-B virus (HBV); Hepatitis-C virus (HCV); Hepatitis-E virus (HEV); Dengue virus (DENV); West Nile virus (WNV).

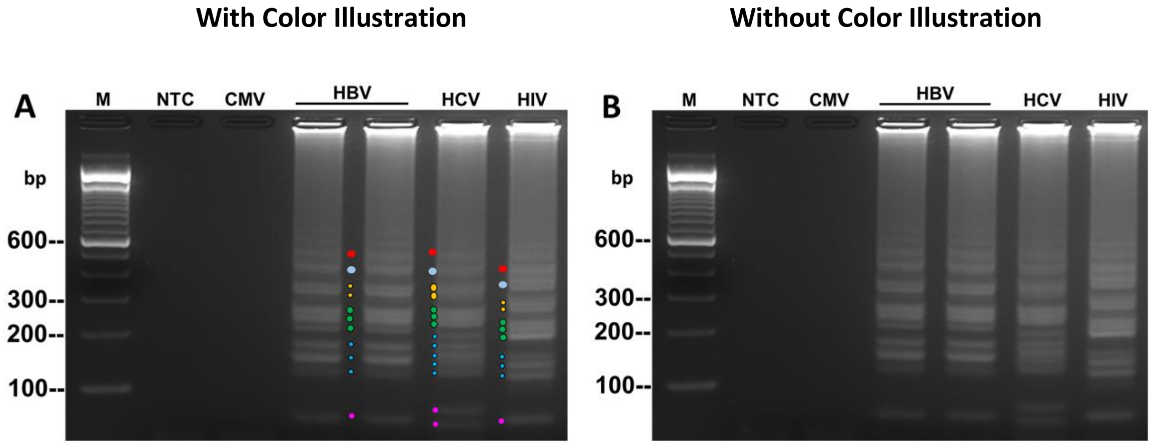


**Supplementary Figure 2. Identification of amplified targets, HBV, HCV, and HIV by banding-pattern. (A)** Gel electrophoretic results demonstrating distinctive ladder-like banding-patterns of amplified products (HBV, HCV, and HIV) and illustrated by color-dots as in Figure S1, S3 and S4. **(B)** Identical gel showing banding patterns without color illustration. Note the fine resolution of the bands, and the uniqueness and consistency in the patterns of the different targets. Gel was run for (circa 50 minutes) to retain the pink-doted differentiating bands below bp 100 (actual bands, not primer-dimers). M = 100 bp marker; NTC = non-template control; CMV = cytomegalovirus; Human Hepatitis-B virus (HBV); Hepatitis-C virus (HCV); Immunodeficiency virus (HIV).

**With Color Illustration** **Without Color Illustration**

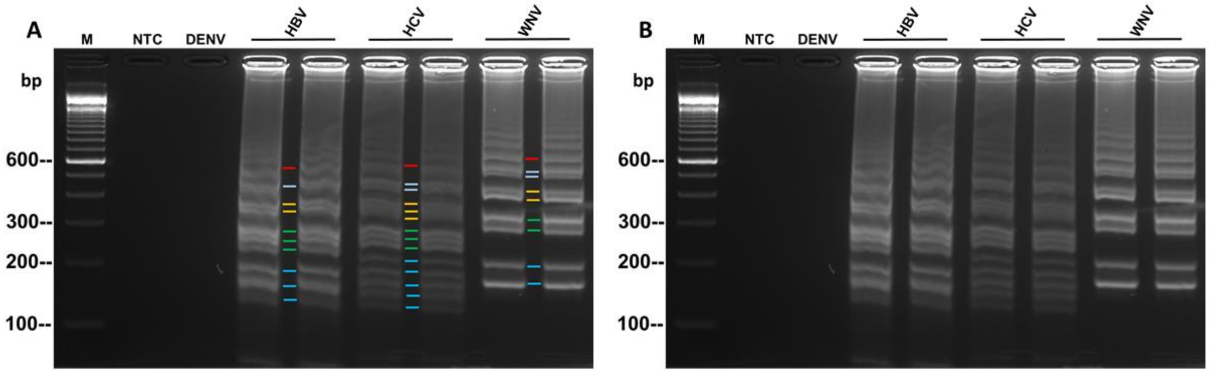


**Supplementary Figure S3. Identification of amplified targets, HBV, HCV, and WNV by banding-pattern.** Electrophoretic analysis showing distinctive ladder-like banding-patterns of amplified products (HBV, HCV, and WNV) in duplicate. **(A)** Figure with color illustration and **(B)** figure without color illustration. **(i)** Note the pattern of bands located between bp 200 and 100 and illustrated by the blue color lines. In this group, HBV has 3 large bands, HCV has about 5, while WNV has 2 bands. The laddering of the HCV pattern extends above that of HBV; HCV has 3 more bands than WNV. **(ii)** In the group of pattern indicated by green lines, HBV and HCV both have 3 bands each with the HCV bands extending above those of HBV, but below the 2 bands of WNV in that pattern. **(iii)** In the group of pattern indicated by yellow lines around bp 300, HBV has 2 bands, HCV has 3 bands with the HCV bands extending above those of HBV, while WNV has only 2 bands. This Gel was run longer (circa 60 minutes) to further separate and resolved the banding-patterns. M = 100 bp marker; NTC = non-template control; Dengue virus (DENV); Hepatitis-B virus (HBV); Hepatitis-C virus (HCV); Hepatitis-E virus (HEV); West Nile virus (WNV).

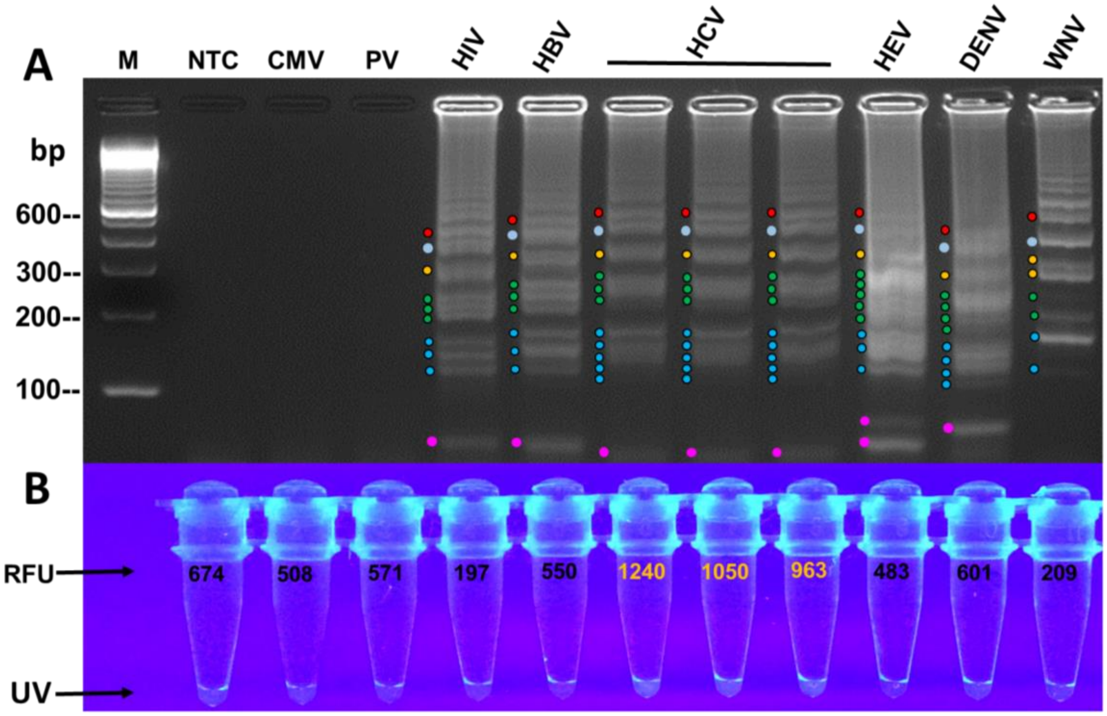


**Supplementary Figure S4. Specific detection by fluorescence and Identification of amplified targets by banding-pattern. (A)** Electrophoretic results showing distinctive ladder-like banding-patterns of amplification products (HIV, HBV, HCV, HEV, DENV, and WNV). Color dots illustrate the difference in grouping of bands, pattern formation, number and sizes of bands, and spacing between patterns (see also Figure S1). **(B)** Fluorescence detection of HCV by the HCV-specific flurooligonucleotide. The results reveals higher RFU and intense fluorescence in the detected HCV samples. M = 100 bp marker; NTC = non-template control; CMV = cytomegalovirus; PV = parvovirus; Human Immunodeficiency virus (HIV); Hepatitis-B virus (HBV); Hepatitis-C virus (HCV); Hepatitis-E virus (HEV); Dengue virus (DENV); West Nile virus (WNV); RFU = Relative Fluorescence Units; UV = ultraviolet naked-eye visualization. Note: This is Figure 4, panel 2 illustrated with color dots.
